# Changes in coronary disease management decisions in real-world practice between 2015 and 2023: insights from the EVAREST/BSE-NSTEP observational study

**DOI:** 10.1093/ehjci/jeaf099

**Published:** 2025-03-22

**Authors:** Casey L Johnson, Samuel Krasner, William Woodward, Emma Mao, Annabelle McCourt, Cameron Dockerill, Katrin Balkhausen, Badrinathan Chandrasekaran, Attila Kardos, Nikant Sabharwal, Soroosh Firoozan, Rizwan Sarwar, Roxy Senior, Rajan Sharma, Kenneth Wong, Daniel X Augustine, Maria Paton, Jamie O’Driscoll, David Oxborough, Keith Pearce, Shaun Robinson, James Willis, Paul Leeson, the EVAREST/BSE-NSTEP Investigators, Abraheem Abraheem, Sanjay Banypersad, Sadie Bennett, Henry Boardman, Christopher Boos, Sudantha Bulugahapitiya, Jeremy Butts, Duncan Coles, Joanna d'Arcy, Jacob Easaw, Sarah Fairbairn, Patrick Gibson, Haytham Hamdan, Shahnaz Jamil-Copley, Gajen Kanaganayagam, Guy Lloyd, Ioannis Moukas, Tom Mwambingu, Thuraia Nageh, Antonis Pantazis, Alexandros Papachristidis, Ronak Rajani, Muhammad Amer Rasheed, Naveed A Razvi, Sushma Rekhraj, Joban Sehmi, Azeem Sheikh, David P Ripley, Kathleen Rose, Michaela Scheuermann-Freestone, Rebecca Schofield, Ayyaz Sultan, Spiros Zidros

**Affiliations:** Cardiovascular Clinical Research Facility, Division of Cardiovascular Medicine, University of Oxford, Oxford, UK; Cardiovascular Clinical Research Facility, Division of Cardiovascular Medicine, University of Oxford, Oxford, UK; Cardiovascular Clinical Research Facility, Division of Cardiovascular Medicine, University of Oxford, Oxford, UK; Barts and The London School of Medicine and Dentistry, Queen Mary University of London, London, UK; Cardiovascular Clinical Research Facility, Division of Cardiovascular Medicine, University of Oxford, Oxford, UK; Cardiovascular Clinical Research Facility, Division of Cardiovascular Medicine, University of Oxford, Oxford, UK; Cardiovascular Clinical Research Facility, Division of Cardiovascular Medicine, University of Oxford, Oxford, UK; Kings College London, London, UK; Royal Berkshire Hospital, Reading, UK; Great Western Hospital, Swindon, UK; Department of Cardiology, Translational Cardiovascular Research Group, Milton Keynes University Hospital NHS Foundation Trust, Milton Keynes, UK; Faculty of Medicine and Health Sciences, University of Buckingham, Buckingham, UK; Oxford University Hospitals NHS Foundation Trust, Oxford, UK; Wycombe Hospital, High Wycombe, UK; Oxford University Hospitals NHS Foundation Trust, Oxford, UK; Northwick Park Hospital, London, UK; St George’s University Hospitals NHS Foundation Trust, Tooting, UK; Lancashire Cardiac Centre, Blackpool Teaching Hospitals NHS Foundation Trust, Blackpool, UK; Royal United Hospitals, Bath, UK; Leeds Institute of Cardiovascular and Metabolic Medicine, University of Leeds, Leeds, UK; St George’s University Hospitals NHS Foundation Trust, Tooting, UK; Diabetes Research Centre, College of Life Sciences, University of Leicester, Leicester, UK; Research Institute for Sport and Exercise Sciences and the Liverpool Centre for Cardiovascular Science, Liverpool John Moores University, Liverpool, UK; Machester University NHS Foundation Trust, Manchester, UK; Imperial Healthcare NHS Trust, London, UK; Royal United Hospitals, Bath, UK; Cardiovascular Clinical Research Facility, Division of Cardiovascular Medicine, University of Oxford, Oxford, UK

**Keywords:** echocardiography, coronary artery disease, stress echocardiography, invasive coronary angiography

## Abstract

**Aims:**

To assess the real-world impact of updated clinical guidelines and literature on the management of patients undergoing stress echocardiography for the assessment of inducible ischaemia across a national health service.

**Methods and results:**

A total of 13 819 patients from 32 UK hospitals, referred for stress echocardiography between 2015 and 2023, were analysed across two phases: phase 1 (2015–2020) and phase 2 (2020–2023). Follow-up data for 1 year was available for 4920 participants through NHS Digital. Patients in phase 2 were younger and presented with a higher cardiovascular risk profile, although sex distribution remained similar across phases. There was an observed reduction in invasive angiography referrals within 1 year following a positive stress echocardiogram (*P* < 0.01), which appeared to be attributed to changes in the management of patients with moderate ischaemia (3–4 segments; *P* < 0.01). For those who did receive invasive assessment, there were no changes in intervention rate (*P* = 0.27), regardless of ischaemic burden. This trend was most evident in centres performing a higher volume of stress echocardiograms.

**Conclusion:**

Coronary disease management pathways have changed within the UK and fewer patients with moderate ischaemia are undergoing invasive coronary angiography. However, coronary intervention rates are unchanged, suggesting that stress echocardiography is being used to improve patient selection for invasive procedures while minimizing unnecessary referrals. Future work will assess if this reduction in angiography referrals is maintained long term, and if there are any effects on patient outcomes.

## Graphical Abstract

**Graphical Abstract:**
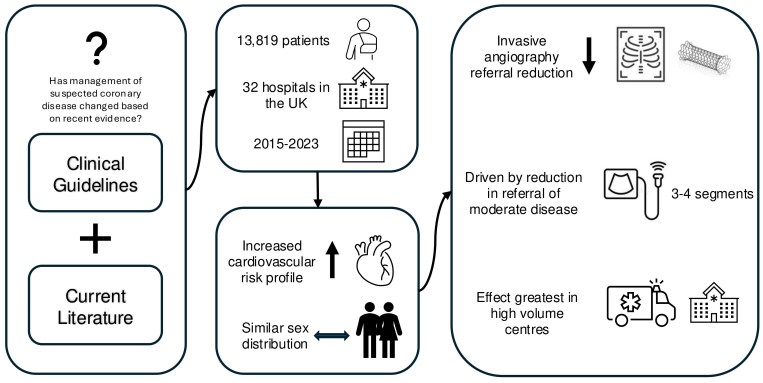

**See the editorial comment for this article ‘Does stress echocardiography change the selection of patients for invasive angiography and revascularization? Insights from the EVAREST/BSE-NSTEP study’, by V. Puntmann and E. Nagel, https://doi.org/10.1093/ehjci/jeaf118.**

## Introduction

For many years, referral for invasive coronary angiography was common for patients with evidence of more than mild cardiac ischaemia on functional imaging to ensure that patients received the opportunity to have revascularization. However, contradicting evidence from randomized studies has led to recent debate over the appropriate investigation and treatment steps in the care pathway for coronary disease.

Several studies have indicated that the risk of death, myocardial infarction, or other cardiac events may not be reduced by an initial invasive strategy in all patients in the non-acute setting,^1–5^ and the ORBITA trial found limited symptomatic benefit of an invasive approach, with no improvement in exercise capacity.^6–9^ However, the ORBITA-2 trial demonstrated improved patient-reported symptom scores for those who received coronary intervention,^10^ and outcome benefits were also evident in long-term follow-up of the ISCHEMIA study^11^ consistent with a recent meta-analysis.^12^

To take account of this emerging data, guidelines have evolved to focus on better selection of high-risk patients for revascularization, preferring an initial medical management strategy in those with lower ischaemic burden. Referral for invasive angiography is reserved for when guideline-directed medical therapy fails to relieve symptoms.^13–15^ Prior to these recent updates, referral of patients for invasive coronary angiography after diagnosis of coronary artery disease was entrained in medical practice, and patients understood this as ‘the best’ way to manage their disease.^16–18^ Whether patients and medical staff would adopt recent guideline updates therefore remained unclear.

Stress echocardiography has been used as a first-line test for patients with chest pain for decades.^14–23^ The Echocardiography Value and Accuracy at Rest and Stress (EVAREST) studied the care pathway of up to 18 000 patients undergoing stress echocardiography across 32 UK hospitals between 2015 and 2023. Recruitment spanned the time that key randomized trials and updated guidelines on cardiovascular disease management were published. As such, the study is uniquely placed to provide insight into whether there have been temporal changes in management decisions for patients presenting with coronary disease in the UK.

## Methods

### Study design

The EVAREST study is a multi-centre observational study evaluating the use, accuracy, and performance of stress echocardiography in real-world practice. From 2020 to 2023, the study incorporated the British Society of Echocardiography National Review of Stress Echocardiography Practice (BSE-NSTEP). The full methodology and 6-month outcome results from the first phase of the study have been previously reported.^24^ The study is registered at ClinicalTrials.gov (NCT03674255), and ethical approval was provided by the Health Research Authority South Central Berkshire Research Ethics Committee (Ethics Reference: 14/SC/1437). The study design predates the common widespread use of patient and public involvement, but patient and public feedback was received via the National Institute for Health and Care Research survey of research participants following the conclusion of the study. Written informed consent was obtained from all patients and the study was conducted in accordance with the Declaration of Helsinki principles.

### Participants

Participants were recruited in two phases with the first recruitment phase running from March 2015 to March 2020 and included patients who were referred for stress echocardiography to assess inducible ischaemia. Recruitment restarted after the initial period of the COVID pandemic and then ran from October 2020 to September 2023, but was expanded to include patients referred to stress echocardiography for any clinical reason. However, only patients referred to stress echocardiography to assess inducible ischaemia were included in this analysis. As there was variation in participating centres between the two phases, only participants recruited at 26 sites that were recruiting centres during both phases of the study were included in this analysis. A subgroup of participants provided consent to link their details with follow-up outcome data provided by NHS England.

### Data collection

Participant demographics and stress echocardiogram procedure details were collected by the local study teams and entered into an electronic database (Castor EDC, Amsterdam, Netherlands). The annual stress echocardiography volume was self-reported by each hospital. Hospital capacity as measured by the number of beds was retrieved from NHS England.^25^ This work uses data provided by patients and collected by the NHS as part of their care and support via the NHS Digital Data Access Request Service. Hospital admission data was collected from the Hospital Episode Statistics Admitted Patient Care database. Data collected included the date and reason for admission, and any procedures undertaken during admission such as invasive coronary angiography, percutaneous coronary intervention, and coronary artery bypass grafting. Reasons for admission were defined by the International Classification of Disease-10th revision coding (ICD-10), and interventions and/or procedures were defined by the OPCS Classification of Interventions and Procedures-4th revision coding (OPCS 4.10) which is the procedural classification used within the NHS in the UK. Data on subsequent diagnostic imaging including invasive coronary angiography was obtained from the Diagnostic Imaging Dataset held by NHS England. Imaging data submitted to NHS England are coded using the Systematized Nomenclature of Medicine-Clinical Terms (SNOMED-CT). Mortality data including date and cause of death were obtained from the Civil Registrations of Death database provided by NHS England. Details of codes used in this analysis are provided in the Supplementary material.

### Statistical analysis

Patient demographics and stress echocardiogram procedural details are reported using standard approaches. Variations in hospital size, measured via annual stress echocardiography volume and hospital bed capacity, were separated into quartiles for comparison. Descriptive statistics were investigated as frequencies and medians [interquartile range (IQR)]. A comparison of discrete data between recruitment phases was conducted using Pearson’s χ^2^ tests. Stress echocardiogram result (positive or negative) was reported as the result by the clinician responsible for each participant’s care. Kaplan–Meier time-to-event curves and log-rank tests were used to assess differences in rates of invasive coronary angiography and percutaneous coronary intervention. Additionally, differences in invasive angiography referrals between recruitment phases were examined according to ischaemic burden (mild: 1–2 ischaemic segments, moderate: 3–4 ischaemic segments, or severe: ≥5 ischaemic segments). Participants with missing data were included in the study, and missing data points were not interpolated. Censored data points were included in all time-to-event analyses to account for any death during the follow-up period. Covariates of age, sex, and demographic variables that were statistically significant between recruitment phases were included in multivariate Cox proportional hazard models. The generated Cox models were used to estimate the hazard ratio (HR) of the temporal recruitment phase as a primary predictor for downstream interventions. Hospital annual stress echocardiogram volume and size (total bed capacity) were included as interaction terms within each Cox model. Sensitivity analyses were conducted via iterative removal of covariates to assess the robustness and reliability of the Cox models. All statistical analysis was carried out using R Statistical Software (v4.4.0, R Core Team 2024), and time-to-event analysis was conducted using the survminer package.

## Results

### Study population

**Figure 1:**
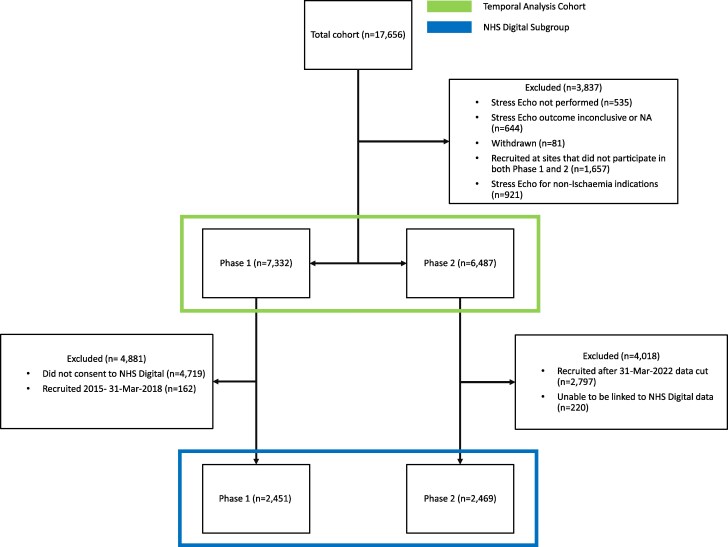
CONSORT diagram illustrating participant recruitment.

Between March 2015 and September 2023, 17 656 patients were recruited into the EVAREST study of which data for 13 819 participants were available for the temporal analysis (7332 in phase 1 and 6487 in phase 2). Follow-up data were received from NHS Digital for a subgroup of 4920 participants (2451 in phase 1 and 2469 in phase 2). Participant inclusion and exclusion for this analysis are described in *Figure 1*.

### Patient demographics and stress echocardiography characteristics (entire cohort)

Full patient demographics are provided in *Table 1*. Median age was similar between phase 1 and phase 2, 66 (IQR 57–73) and 65 (IQR 57–74) years, respectively. Patient sex was also consistent across both phases (56.3 vs. 57.6%), but patients recruited in phase 2 had a higher incidence of hypertension (48.3 vs. 53.4%), hypercholesterolaemia (39.9 vs. 47.9%) and diabetes (18.2 vs. 22.7%) (all *P* < 0.001). The percentage of patients reporting that current smoking practices remained consistent, but there was an increase in ex-smokers (49.7 vs. 54.4%) (*P* < 0.001). There was a decrease in peripheral vascular disease in phase 2 (2.9 vs. 1.5%) as well as a decrease in previous PCI (31.2 vs. 20.5%) (both *P* < 0.001). There were no significant changes in the percentage of previous MI, CABG, and the presence of resting regional wall motion abnormalities between phases.

**Table 1:** Participant demographics

	Phase 1 total cohort (*n* = 7332)	Phase 2 total cohort (*n* = 6487)	*P*-value*	Phase 1 NHS Digital subgroup (*n* = 2451)	Phase 2 NHS Digital subgroup (*n* = 2469)	*P*-value**
Participant demographics						
Male (%)	4126/7332 (56.3)	3739/6486 (57.6)	0.10	1440/2451 (58.8)	1429/2469 (57.9)	0.53
Median age (years) (IQR)	66 (57–73)	65 (57–74)	0.20	67 (59–74)	65 (56–73)	**<0**.**001**
Median BMI (kg/m^2^) (IQR)	28.2 (25.0–31.9)	28.1 (25–31.6)	0.87	27.9 (24.9–31.5)	28.1 (24.9–31.9)	**<0**.**05**
Positivity rate (%)	1394/7332 (19.0)	1231/6487 (19.0)	0.96	448/2451 (18.3)	523/2469 (21.2)	**<0**.**05**
Current smoker (%)	860/7045 (12.2)	744/6285 (11.8)	0.63	203/2541 (8.3)	374/2469 (15.1)	**<0**.**001**
Ex-smoker (%)	2687/7045 (38.1)	2125/6285 (33.8)	**<0**.**001**	1025/2451 (41.8)	786/2469 (31.8)	**<0**.**001**
Non-smoker (%)	3498/7045 (49.7)	3416/6285 (54.4)	**<0**.**001**	1144/2451 (46.7)	1242/2469 (50.3)	**<0**.**05**
Hypertension (%)	3346/6931 (48.3)	3445/6455 (53.4)	**<0**.**001**	1332/2295 (58.0)	1301/2466 (52.8)	**<0**.**001**
Hypercholesterolaemia (%)	2767/6931 (39.9)	3090/6455 (47.9)	**<0**.**001**	1064/2295 (46.4)	1187/2466 (48.1)	0.22
Diabetes mellitus (%)	1331/7332 (18.2)	1463/6455 (22.7)	**<0**.**001**	506/2451 (20.6)	525/2466 (21.3)	0.57
Peripheral vascular disease (%)	204/6931 (2.9)	99/6453 (1.5)	**<0**.**001**	74/2295 (3.2)	40/2467 (1.6)	**<0**.**001**
Previous MI (%)	1243/7214 (17.2)	1121/6453 (17.4)	0.83	445/2423 (18.4)	472/2467 (19.1)	0.49
Previous PCI (%)	2256/7228 (31.2)	1323/6453 (20.5)	**<0**.**001**	835/2425 (34.4)	548/2467 (22.2)	**<0**.**001**
Precious CABG (%)	529/7241 (7.3)	441/6453 (6.8)	0.28	178/2429 (7.3)	156/2467 (6.3)	0.16
Resting RWMA (%)	1080/7328 (14.7)	916/6449 (14.2)	0.37	397/2449 (16.2)	313/2467 (12.7)	**<0**.**001**
Stress echocardiogram details						
Exercise (%)	2211/7330 (30.2)	2508/6480 (38.7)	**<0**.**001**	778/2451 (31.7)	982/2469 (39.8)	**<0**.**001**
Pacemaker (%)	11/7332 (0.2)	39/6480 (0.6)	**<0**.**001**	7/2451 (0.3)	18/2469 (0.7)	**<0**.**05**
Dobutamine (%)	5108/7330 (69.7)	3919/6480 (60.5)	**<0**.**001**	1665/2451 (67.9)	1464/2469 (59.3)	**<0**.**001**
Atropine use in DSE (%)	2518/5091 (49.5)	1773/3859 (45.9)	**<0**.**001**	734/1665 (44.1)	689/1464 (47.1)	0.10
Contrast used	5258/7309 (71.9)	5279/6407 (82.4)	**<0**.**001**	1961/2440 (80.4)	1907/2444 (78.0)	**<0**.**05**
SonoVue (%)	4847/7309 (66.3)	4193/6407 (65.4)	0.07	1729/2440 (70.9)	1668/2444 (68.2)	<0.05
Luminity (%)	393/7309 (5.4)	1080/6407 (16.9)	**<0**.**001**	220/2440 (9.0)	236/2444 (9.7)	0.44
Other (e.g. Optison) (%)	18/7309 (0.2)	6/6407 (0.1)	**0**.**031**	12/2440 (0.5)	3/2444 (0.1)	**<0**.**05**

Bold values indicate statistically significant.

^*^ *P*-value comparison between recruitment phases (overall cohort).

^**^ *P*-value comparison between recruitment phases (NHS Digital subgroup).

Stress echocardiogram positivity was similar between phases at 19.0%. While dobutamine remained the most common stressor used between phases, there was a decrease from 69.7% in phase 1 to 60.5% in phase 2 (*P* < 0.001). This saw a corresponding increase in the use of exercise stress from 30.2 to 38.7% between phases (*P* < 0.001). There was also a marginal increase in the use of pacemaker stress (0.2 vs. 0.6%, *P* < 0.001). Within dobutamine stress echocardiograms, there was a decrease in the use of atropine (49.5 vs. 45.9%, *P* < 0.001). The use of contrast increased (71.9 vs. 82.4%, *P* < 0.001) with a related increase in the use of Luminity as a contrast agent (5.4 vs. 16.9%, *P* < 0.001). Patient demographics and stress echocardiogram procedural details separated by stress echocardiogram outcome are provided in the Supplementary material.

### Patient demographics and stress echocardiography characteristics (NHS Digital subgroup)

The NHS Digital subgroup had similar demographics, medical history, and stress echocardiography practice as the overall cohort except for a marginally higher stress echocardiogram positivity rate in phase 2 (18.3 vs. 21.2%, *P* < 0.05) despite a lower prevalence of resting regional wall motion abnormalities (16.2 vs. 12.7%, *P* < 0.001). Comorbidity was similar to the overall cohort with the exception of less hypertension in phase 2 (58.0 vs. 52.8%), peripheral vascular disease (3.2 vs. 1.6%), and a higher rate of current smokers (8.3 vs. 15.1%) (all *P* < 0.001) (*Table 1*).

### Participant management

**Figure 2:**
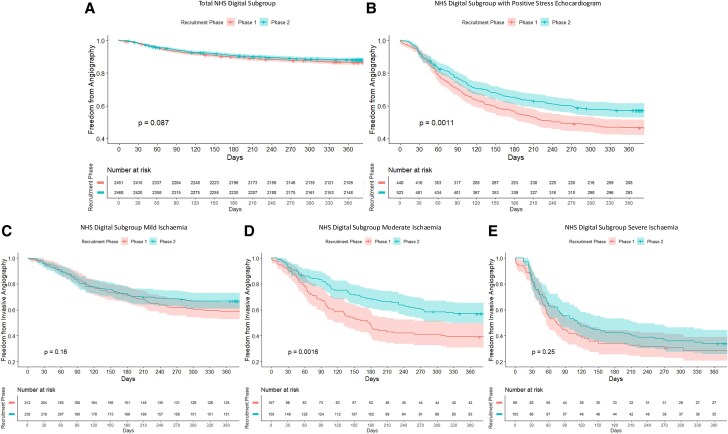
Kaplan–Meier analysis of freedom from invasive coronary angiogram total NHS Digital subgroup (*A*), those with a positive stress echocardiogram (*B*), and according to ischaemic burden of mild, moderate, and severe (*C–E*).

Time-to-event analysis within the NHS Digital subgroup is provided in *Figure 2*, showing no difference in invasive angiography referral rate between groups in the total subgroup analysis (*Figure 2A*), but a decrease in referral to invasive coronary angiography within 1 year for participants with a positive stress echocardiogram result in phase 2 compared with phase 1 (*P* < 0.01, *Figure 2B*). Analysis of invasive angiography referral rate according to ischaemic burden demonstrated the main reduction in referral rates was seen in participants with moderate ischaemia with no significant difference in participants with mild or severe ischaemia (*P* < 0.01) (*Figure 2C–E*).

As reported in *Table 2*, there was a reduced HR for invasive angiography in phase 2 participants within the whole NHS Digital subgroup analysis, HR 0.77 (95% CI 0.66–0.91, *P* < 0.01) and in those with a positive stress echocardiogram, HR 0.75 (95% CI 0.62– 0.92, *P* < 0.01) after covariate adjustment. There was no significant difference in the number of percutaneous coronary interventions performed in those with a positive stress echocardiogram (*P* = 0.27), regardless of ischaemic burden. As shown in *Table 3*, hospitals with an annual stress echocardiography volume of <400 had a relative increase in the proportion of invasive angiogram referrals from phase 1 to phase 2 (14.4 vs. 21.5%), resulting in an inverse association between HR for invasive angiography and volume of stress echocardiograms performed in a centre following a positive stress echocardiogram, HR 0.71 (95% CI 0.64– 0.79, *P* < 0.001) (*Table 2*).

**Table 2:** Multivariate Cox regression analysis of invasive coronary angiography in NHS Digital subgroup

Variable	Total subgroup			Positive stress echocardiogram		
	HR	95% CI	*P*-value	HR	95% CI	*P*-value
Recruitment phase (phase 2)	0.77	0.65–0.91	**<0**.**01**	0.75	0.62–0.92	**<0**.**01**
Age	0.99	0.99–1.00	0.08	0.99	0.98–1.00	**<0**.**05**
Sex (male)	1.16	0.97–1.38	0.10	1.16	0.94–1.42	0.17
SE outcome (positive)	17.38	14.29–21.15	**<0**.**001**	—	—	—
Current smoker	0.90	0.70–1.16	0.41	0.87	0.65–1.16	0.35
Hypertension	1.19	1.00–1.41	**<0**.**01**	1.16	0.95–1.41	0.14
Previous peripheral vascular disease	0.91	0.56–1.48	0.71	1.11	0.67–1.84	0.69
Previous PCI	0.79	0.66–0.96	**<0**.**05**	0.73	0.59–0.90	**<0**.**01**
Baseline RWMA	1.21	1.00–1.46	**<0**.**05**	1.12	0.91–1.37	0.29
Hospital demographics						
SE per year (per quartile)	0.75	0.68–0.81	**<0**.**001**	0.71	0.64–0.79	**<0**.**001**
Bed number (per quartile)	1.10	1.00–1.21	**<0**.**05**	1.12	1.00–1.25	0.06

Bold values indicate statistically significant.

**Table 3:** Invasive angiography referrals relative to hospital volume and capacity in NHS Digital subgroup

	Total angiograms (%)*	Angiogram phase 1 (%)*	Angiogram phase 2 (%)*
Stress echocardiography volume			
<400	137 (17.3)	68 (14.4)	69 (21.5)
400–599	116 (10.5)	72 (12.4)	44 (8.4)
600–850	102 (13.0)	44 (13.5)	58 (12.6)
>850	264 (11.8)	145 (13.5)	119 (10.2)
Hospital capacity (number of beds)			
<600	194 (14.9)	104 (14.3)	90 (15.8)
600–799	84 (12.0)	74 (14.5)	10 (5.3)
800–1000	155 (10.4)	35 (10.0)	120 (10.6)
>1000	186 (12.9)	116 (13.5)	70 (12.2)

^*^Percentage of recruitment contribution.

## Discussion

This study provides real-world data on the changing management pathways of patients who are referred for stress echocardiography for the assessment of myocardial ischaemia. This study benefits from having a large patient population from a wide range of recruiting centres across the UK, increasing the diversity of the data and generalizability of the results. There is an overall reduction in the referral to invasive coronary angiography following a positive stress echocardiogram, presumably in favour of medical therapy. The observed reduction in angiography referrals appears to be driven by a shift in management in patients with moderate (3–4 segments) myocardial ischaemia.

These findings support recent clinical trial results which reported no additional short-term risk from an initial medical management strategy in patients with stable chest pain.^5,6,9^ This data also supports recommendations in the updated 2024 ESC guidelines suggesting that patients receive medical therapy following confirmation of diagnosis with first-line testing. Invasive angiography and possible revascularization are then suggested for patients who are categorized as high risk following diagnostic testing, or who continue to have symptoms despite optimal medical therapy.^15^ While it has been reported that invasive coronary angiography use is reduced with the use of non-invasive CT angiography for chest pain,^26^ the current study would suggest that this is a function of using an imaging test rather than providing information on anatomy or function. Patients included in this analysis did not undergo CT angiography prior to the stress echocardiogram and there is no evidence patients were being referred for CT angiography instead of invasive angiography after the test. This may be due to issues with access to CT angiography in the UK.^27^ Thus, the reduction in angiography referrals observed is more likely to be attributed to a change in decisions about management approach.

Why hospitals performing a low volume of stress echocardiograms, i.e. < 400 per year, did not appear to demonstrate this drop in referral pattern needs further consideration. Large volume centres may more rapidly adapt the practice to include guideline updates or there may be differences in patient referral patterns or disease severity between centres that were not captured within the datasets available in this analysis. Alternatively, previously reported data from the EVAREST study illustrating associations of ischaemic burden and outcomes^24^ may have also influenced patient management workflow at participating centres. Nevertheless, this pattern appears consistent when using different metrics of centre size such as hospital bed number.

Interestingly, rates of percutaneous coronary intervention remain consistent across phases, and this likely reflects the use of coronary intervention largely in those identified with severe ischaemia, in whom referral rates have remained consistent.^1,9^ Therefore, while this analysis reveals a shift in current practice, it also provides evidence that a more selective approach for use of angiography is not reducing the rate of intervention within patients with coronary disease.^28,29^ An analysis of 5-year outcomes for a subgroup of the EVAREST cohort has recently been published indicating that a positive stress echocardiogram, and degree of ischaemic burden, is associated with an increased risk of both all cause and cardiac-related mortality, as well as myocardial infarction, and predicts the need for revascularization.^30^ As this analysis relied on outcomes over 5 years, this primarily reflects outcomes of the referral practice in the first phase of EVAREST. Future long-term follow-up, up to 10 years, will provide an opportunity to investigate whether outcomes remain similar in the second phase of EVAREST.

There are limitations to this analysis. Firstly, due to the nature of the data collection, there are no results on patient symptoms throughout the management period. Some studies have shown that patient-reported symptoms and quality of life are improved with an invasive management strategy even when a reduction in mortality and adverse events is not identified.^5,10^ However, this improvement in symptoms appears inconsistent^6^ and some investigators have attributed this to a placebo effect.^31^ Secondly, the time horizon used in this analysis is limited to 1 year. Most patients referred for an elective coronary angiogram following their stress echocardiogram will be seen within this timeframe and this appears appropriate to account for further investigational testing such as invasive angiography. However, any invasive angiography performed more than 1 year following uncontrolled symptoms with an initial medical management strategy may be unaccounted for. Thirdly, the study was focused on an evaluation of real-world practice and it is possible associations may differ if other stress echocardiography protocols were applied in practice that used additional measures that may improve predictive accuracy such as heart rate reserve. Fourthly, it should be noted that data received from the data request service from NHS Digital has inherent limitations. If no outcome data was received after supplying NHS Digital with participant identifiers for data linkage, it was assumed that this participant had no follow-up outcomes or events within the requested timeframe. This could, however, also mean that the participant had follow-up data, but was not able to be retrieved by NHS Digital for unknown reasons. Fifthly, while sites remained consistent for the temporal analysis, not all sites began recruiting at the same time and had varying recruitment rates. Therefore, some sites contributed more proportionally to the dataset. Finally, due to the nature of the prospective consented study design, there may be a selection bias among those enrolled towards those with an interest in research participation.

## Conclusion

This study provides real-world evidence of a change in coronary disease management decisions within the NHS. Since 2020, there has been a small but significant reduction in the number of patients who are referred for invasive angiography after a positive stress echocardiogram. This can be attributed to a reduced referral to invasive angiography in patients with moderate ischaemia, while those with mild and severe disease have not experienced significant changes in their management pathways. Interestingly, rates of use of percutaneous coronary intervention did not change over the recruitment period, suggesting a better selection of patients for angiography. These results should be considered in the context of the sample size and time horizon, and future work will aim to further confirm these management changes, and establish what effect, if any, this has on patient outcomes long term.

## Supplementary Material

jeaf099_Supplementary_Data

## Data Availability

Data on patient demographics and stress echocardiogram procedures will be made available upon reasonable request. Outcome data collected via NHS Digital is not available for sharing.

## EVAREST/BSE-NSTEP Investigators

Abraheem Abraheem^19^, Sanjay Banypersad^20^, Sadie Bennett^21^, Henry Boardman^7^, Christopher Boos^22^, Sudantha Bulugahapitiya^23^, Jeremy Butts^24^, Duncan Coles^25^, Joanna d'Arcy^8^, Jacob Easaw^13^, Sarah Fairbairn^26^, Patrick Gibson^27^, Haytham Hamdan^28^, Shahnaz Jamil-Copley^29^, Gajen Kanaganayagam^30^, Guy Lloyd^31^, Ioannis Moukas^32^, Tom Mwambingu^33^, Thuraia Nageh^34^, Antonis Pantazis^35^, Alexandros Papachristidis^36^, Ronak Rajani^37^, Muhammad Amer Rasheed^38^, Naveed A. Razvi^39^, Sushma Rekhraj^29^, Joban Sehmi^40^, Azeem Sheikh^22^ David P. Ripley^41^, Kathleen Rose^42^, Michaela Scheuermann-Freestone^43^, Rebecca Schofield^44^, Ayyaz Sultan^28^, Spiros Zidros^45^

^19^Department of Cardiology, Tameside and Glossop Integrated Care NHS Foundation Trust, Ashton-under-Lyne, UK.

^20^Department of Cardiology, Royal Blackburn Hospital, East Lancashire Hospitals NHS Trust, Burnley, UK.

^21^Department of Cardiology, Heart & Lung Centre, Royal Stoke University Hospital, University Hospitals of North Midlands NHS Foundation Trust, Stoke, UK.

^22^Department of Cardiology, Poole Hospital NHS Foundation Trust, Poole, UK.

^23^Department of Cardiology, Bradford Royal Infirmary Hospital, Bradford Teaching Hospitals NHS Foundation Trust, Bradford, UK.

^24^Department of Cardiology, the Calderdale Royal Hospital and Huddersfield Royal Infirmary, Calderdale and Huddersfield NHS Foundation Trust, Calderdale, UK.

^25^Department of Cardiology, Broomfield Hospital, Mid and South Essex NHS Foundation Trust, Broomfield, UK.

^26^Bristol Heart Institute, University Hospitals Bristol and Weston NHS Foundation Trust, Bristol, UK.

^27^Royal Infirmary Edinburgh and Western General Hospital, NHS Lothian, UK.

^28^Department of Cardiology, Wrightington, Wigan and Leigh NHS Foundation Trust, Wigan, UK.

^29^Department of Cardiology, Nottingham University Hospitals NHS Trust, Nottingham, UK.

^30^Department of Cardiology, West Middlesex University Hospital, Chelsea and Westminster Hospital NHS Foundation Trust, London, UK.

^31^University College London, Barts Health NHS Trust, UK.

^32^Department of Cardiology, Warrington and Halton Teaching Hospitals NHS Foundation Trust, Warrington, UK.

^33^Department of Cardiology, The Mid Yorkshire Hospitals NHS Trust, Pinderfields, UK.

^34^Department of Cardiology, Southend University Hospital, Mid and South Essex NHS Foundation Trust, Southend-on-Sea, UK.

^35^Department of Cardiology, North Middlesex University Hospital NHS Trust, London, UK.

^36^Department of Cardiology, King's College Hospital NHS Foundation Trust, London, UK.

^37^Department of Cardiology, Guy's and St Thomas' NHS Foundation Trust, London, UK.

^38^Department of Cardiology, Yeovil District Hospital, Somerset NHS Foundation Trust, Yeovil, UK.

^39^Department of Cardiology, East Suffolk and North Essex NHS Foundation Trust, Ipswich, UK.

^40^West Hertfordshire Hospitals NHS Trust, UK.

^41^Department of Cardiology, Northumbria Healthcare NHS Foundation Trust, North Tyneside, UK.

^42^Department of Cardiology, Northampton General Hospital NHS Trust, Northampton, UK.

^43^Department of Cardiology, Hampshire Hospitals NHS Foundation Trust, Basingstoke, UK.

^44^Department of Cardiology, North West Anglia NHS Foundation Trust, Peterborough, UK.

^45^Department of Cardiology, Bedford Hospital, Bedfordshire Hospitals NHS Foundation Trust, Bedford, UK.
