# Supplementary material for: Changes in coronary disease management decisions in real-world practice between 2015 and 2023: insights from the EVAREST/BSE-NSTEP observational study
Source: Eur Heart J Cardiovasc Imaging. 2025 Mar 22;26(7):1099–106. doi: 10.1093/ehjci/jeaf099 (PMC12206575; doi:10.1093/ehjci/jeaf099)
Supplement: jeaf099_Supplementary_Data [file jeaf099_supplementary_data.zip › Supplementary Table 1.docx]

**Supplementary Table 1:** Participant demographics and stress echocardiogram details for those with a positive stress echocardiogram in total cohort.

|  | Phase 1  (n=1394) | Phase 2  (n=1231) | p-value |
| --- | --- | --- | --- |
| **Participant Demographics** |  |  |  |
| Male (%) | 901/1394 (64.6) | 837/1231 (68.0) | 0.05 |
| Median age (years) (IQR) | 68 (10.21) | 67 (10.30) | 0.55 |
| Median BMI (kg/m2) (IQR) | 28.6 (5.2) | 28.11 (5.4) | 0.35 |
| Current smoker (%) | 177/1344 (13.2) | 158/1178 (13.4) | 0.92 |
| Ex-smoker (%) | 540/1344 (40.2) | 420/1178 (35.7) | **<0.05** |
| Non-smoker (%) | 627/1344 (46.7) | 600/1178 (50.9) | 0.05 |
| Hypertension (%) | 716/1341 (53.4) | 727/1223 (59.4) | **<0.01** |
| Hypercholesterolaemia (%) | 664/1394 (49.5) | 651/1223 (53.2) | 0.06 |
| Diabetes mellitus (%) | 308/1394 (22.1) | 376/1223 (30.7) | **<0.001** |
| Peripheral vascular disease (%) | 57/1341 (4.3) | 22/1225 (1.8) | **<0.001** |
| Family history of premature CAD (%) | 64/1394 (4.8) | 445/1223 (36.4) | **<0.001** |
| Previous MI (%) | 354/1394 (25.9) | 270/1231 (22.0) | 0.02 |
| Previous PCI (%) | 578/1371 (42.2) | 321/1225 (26.2) | **<0.001** |
| Previous CABG (%) | 197/1373 (14.3) | 142/1225 (11.6) | **<0.05** |
| Resting RWMA (%) | 497/1393 (35.7) | 327/1227 (26.7) | **<0.001** |
|  |  | | |
| **Stress Echocardiogram Details** |  |  |  |
| Exercise (%) | 387/1394 (27.8) | 341/1230 (27.7) | 0.972 |
| Pacemaker (%) | 5/1394 (0.40) | 17/1230 (1.4) | **<0.01** |
| Dobutamine (%) | 1002/1394 (71.9) | 871/1230 (70.8) | 0.53 |
| Atropine use in DSE (%) | 533/1002 (53.1) | 438/871 (50.3) | 0.241 |
| Contrast used | 1010/1390 (72.7) | 1098/1219 (90.0) | **<0.001** |
| SonoVue (%) | 942/1390 (67.8) | 953/1219 (78.2) | **<0.001** |
| Luminity (%) | 63/1390 (4.5) | 144/1219 (11.8) | **<0.001** |
| Other (e.g. Optison) (%) | 5/1390 (0.40) | 1/1219 (0.10) | 0.14 |
| No contrast (%) | 380/1390 (27.3) | 121/1219 (9.9) | **<0.001** |
