# Supplementary material for: Changes in coronary disease management decisions in real-world practice between 2015 and 2023: insights from the EVAREST/BSE-NSTEP observational study
Source: Eur Heart J Cardiovasc Imaging. 2025 Mar 22;26(7):1099–106. doi: 10.1093/ehjci/jeaf099 (PMC12206575; doi:10.1093/ehjci/jeaf099)
Supplement: jeaf099_Supplementary_Data [file jeaf099_supplementary_data.zip › Supplementary Table 2.docx]

|  | Phase 1  (n=5938) | Phase 2  (n=5255) | p-value |
| --- | --- | --- | --- |
| **Participant Demographics** |  |  |  |
| Male (%) | 3225/5938 (54.3) | 2902/5255 (55.2) | 0.33 |
| Median age (years) (IQR) | 65 (11.87) | 65 (12.22) | 0.26 |
| Median BMI (kg/m2) (IQR) | 28.1 (5.6) | 28.1 (5.6) | 0.80 |
| Current smoker (%) | 583/5938 (11.5) | 586/5256 (11.1) | 0.56 |
| Ex-smoker (%) | 2147/5938 (36.2) | 1705/5256 (32.4) | **<0.001** |
| Non-smoker (%) | 2871/5938 (48.3) | 2816/5256 (53.6) | **<0.001** |
| Hypertension (%) | 2630/5938 (47.0) | 2718/5232 (51.9) | **<0.001** |
| Hypercholesterolaemia (%) | 2103/5590 (37.6) | 2439/5232 (46.6) | **<0.001** |
| Diabetes mellitus (%) | 1023/5938 (17.2) | 1087/5232 (20.8) | **<0.001** |
| Peripheral vascular disease (%) | 147/5590 (2.6) | 77/5228 (1.5) | **<0.001** |
| Family history of premature CAD (%) | 400/5938 (7.2) | 1774/5232 (33.9) | **<0.001** |
| Previous MI (%) | 889/5938 (15.2) | 851/5256 (16.3) | 0.123 |
| Previous PCI (%) | 1678/5938 (28.6) | 1002/5228 (19.2) | **<0.001** |
| Previous CABG (%) | 332/5938 (5.7) | 299/5228 (5.7) | 0.89 |
| Resting RWMA (%) | 583/5938 (9.8) | 589/5222 (11.3) | **<0.01** |
|  |  | | |
| **Stress Echocardiogram Details** |  |  |  |
| Exercise (%) | 1824/5936 (30.7) | 2167/5250 (41.3) | **<0.001** |
| Pacemaker (%) | 6/5936 (0.10) | 22/5250 (0.40) | **<0.001** |
| Dobutamine (%) | 4106/5936 (69.2) | 3048/5250 (58.1) | **<0.001** |
| Atropine use in DSE (%) | 2001/4106 (48.7) | 1356/3048 (44.5) | **<0.001** |
| Contrast used | 4284/5919 (71.8) | 4181/5188 (80.1) | **<0.001** |
| SonoVue (%) | 3905/5919 (66.0) | 3240/5188 (62.5) | **<0.001** |
| Luminity (%) | 330/5919 (5.6) | 936/5188 (18.0) | **<0.001** |
| Other (e.g. Optison) (%) | 13/5919 (0.20) | 5/5188 (0.10) | 0.10 |
| No contrast (%) | 1671/5919 (28.2) | 1007/5188 (19.4) | **<0.001** |

**Supplementary Table 2:** Participant demographics and stress echocardiogram details for those with a negative stress echocardiogram in total cohort.
